# Synergistic effect of Chaihu-Shugan-San combined with paroxetine tablets in the treatment of acquired premature ejaculation: study protocol for a randomized controlled proof-of-concept trial

**DOI:** 10.3389/fphar.2025.1651654

**Published:** 2025-10-07

**Authors:** Shengqiang Qian, Jintao Wei, Peihai Zhang, Ziyang Ma, Yu Guo, Feiqiang Ren, Wei Xiong

**Affiliations:** ^1^ Department of Urology, Chongqing Hospital of Traditional Chinese Medicine, Chongqing, China; ^2^ The Urology and Andrology Department, Hospital of Chengdu University of Traditional Chinese Medicine, Chengdu, Sichuan, China

**Keywords:** premature ejaculation, traditional Chinese medicine, paroxetine, magnetic resonance imaging, brain

## Abstract

**Introduction:**

Acquired premature ejaculation (APE) is a common male sexual dysfunction characterized by a clinically significant reduction in intravaginal ejaculatory latency time. In China, its prevalence ranges from 4% to 39%, while globally, approximately 26% of men report premature ejaculation (PE), with APE accounting for 18.8% of cases. The mechanisms of APE remain unclear, with psychological factors (e.g., anxiety, depression) and neurological factors identified as key influences. Selective serotonin reuptake inhibitors (SSRIs), such as paroxetine, delay ejaculation by elevating synaptic 5-HT levels, but they are associated with side effects and poor compliance. Chaihu-Shugan-San (CHSGS), a classical Traditional Chinese Medicine (TCM) formula for depression and anxiety, is commonly used in TCM andrology for APE. It is hypothesized that CHSGS alleviates APE symptoms by improving emotional states through central nervous system modulation.

**Methods and analysis:**

This is a randomized, blinded interventional trial with four parallel groups. A total of 84 eligible participants will be randomly assigned to one of four groups: the paroxetine group, CHSGS group, CHSGS + paroxetine group, or placebo group. The primary outcomes are Intravaginal ejaculatory latency time (IELT) and Pre- and post-treatment brain fMRI scans. The Secondary Outcomes include Premature ejaculation profile (PEP), Chinese Index of Premature Ejaculation (CIPE) and TCM syndrome score.

**Ethics and dissemination:**

The trial has been registered with the International Traditional Medicine Clinical Trial Registry (Registration No. ITMCTR2025000309) and Ethics Committee of Chongqing Hospital of Traditional Chinese Medicine (Registration No. 2023-ky-87). It will be proceeded in the Departments of Urology and Radiology at Chongqing Traditional Chinese Medicine Hospital in Chongqing, China.

**Trial registration number:**

ITMCTR2025000309.

## Strengths and limitations of this study

Focuses on central nervous mechanisms of acquired PE using fMRI, investigating how CHSGS may improve symptoms via CNS modulation, addressing gaps in APE neurobiology research.

Combines clinical metrics (IELT) and fMRI to evaluate both behavioral and central nervous system mechanisms, with secondary measures including PE-specific scales and TCM syndrome scores.

Uses daily paroxetine instead of on-demand dapoxetine, potentially affecting comparability due to different administration modes.

CHSGS is a multi-ingredient formula, making it difficult to isolate active components or single targets, limiting mechanistic analysis to macro-level brain activity changes.

## Introduction

Premature ejaculation (PE) is a prevalent form of male sexual dysfunction, characterized by ejaculation occurring earlier than desired during vaginal sexual intercourse. In general, we categorize premature ejaculation into two types: lifelong PE, which occurs every time or almost every time from the first sexual experience onwards, and acquired PE (APE), which develops after a period of sexual activity without any ejaculation issues. In APE, there is a clinically significant and bothersome reduction in the time to ejaculation, often reduced to 3 min or less (Salonia et al., 2021). Among the Chinese population, the prevalence of PE ranges from around 4%–39%. Globally, about 26.0% of men report experiencing PE, with APE making up 18.8% of these cases (Yang et al., 2023). Due to embarrassment and lack of awareness, the possible prevalence of PE is generally underestimated, and men with PE often experience severe psychological difficulties, avoid physical and emotional intimacy, and fall victim to false medical advertisements and unproven medical management (Shindel et al., 2025).

The mechanism of PE is currently unclear, and psychological factors are an important influence, but there is growing evidence that neurological factors are a complex influence (Yuan et al., 2025), and studies in animal models and with clinical experience have found that neurological factors are one of the most important factors in premature ejaculation (Aru et al., 2023). The brain expresses rich and dynamic patterns of neural activity across multiple spatial and temporal scales (Gul et al., 2022), and the developmental process of PE also involves multiple brain regions and systems, with complex and highly coordinated relationships among them (Wu et al., 2024). Previous animal studies have proposed a central control mechanism for ejaculatory dysfunction (Liu et al., 2025), It is currently believed that the possible neural mechanisms of PE are due to dysfunction of somatosensory centers and dysregulation of neurophysiological aspects (Ma et al., 2019).

Selective serotonin reuptake inhibitors, such as dapoxetine and paroxetine, have been shown to delay ejaculation. Paroxetine, as a selective 5-hydroxytryptamine (5-HT) reuptake inhibitor, can regulate central nervous activity by elevating the level of 5-HT in the synaptic gap, inhibit the ejaculatory reflex from the spinal cord level, improve the control of ejaculation, and prolong the ejaculatory latency period (Raveendran and Agarwal, 2021). Emerging research indicates that paroxetine administration could modulate neurofunctional dynamics in major depressive disorder, particularly influencing amygdala reactivity and functional connectivity patterns within associated neural networks (Tassone et al., 2024). However, these medications carry the risk of side effects and can be expensive. As a result, many patients have poor compliance, which makes it much more difficult to treat the disease (Niu et al., 2024).

Traditional Chinese medicine (TCM) is regarded as an effective treatment for APE. Unlike Western medicine, TCM requires a holistic approach, tailoring treatment based on the patient’s overall physical condition. In a multicenter survey conducted in China, the intervention rate of TCM for PE reached 39.4% (Sansone et al., 2024). Current literature has demonstrated that the occurrence of APE is linked to psychological disorders, particularly anxiety and depression (Yang et al., 2019). Chaihu-Shugan-San (CHSGS) is a classical prescription to treat depression, Several research teams have confirmed through both clinical studies and animal experiments that CHSGS has a significant effect in alleviating depression and anxiety (Zhu et al., 2022; Zhang et al., 2024; Liu et al., 2020). More studies have focused on the mechanisms by which CHSGS improves depression, and current evidence suggests that the antidepressant properties of CHSGS appear to mediate through multiple interrelated biological systems, including monoaminergic neurotransmission, hypothalamic-pituitary-adrenal axis regulation, brain-derived neurotrophic factor, synaptic remodeling mechanisms, gut-brain axis interactions, and inflammatory pathway modulation. The compound likely exerts its therapeutic effects via multimodal mechanisms involving both direct modulation of these core pathophysiological targets and indirect interactions with downstream molecular effectors, such as receptor systems, intracellular signaling cascades, and epigenetic regulators, with predominant actions localized to central neurochemical processes (Guo et al., 2024). In the clinical practice of TCM andrology, this herbal formula is also commonly used in clinical practice for the treatment of APE. Thus, we hypothesized that CHSGS could alleviate symptoms of APE and enhance patients’ quality of life by improving their emotional state though central nervous system.

Therefore, in this protocol, we plan to perform a double-blind randomized controlled trial, aiming to:

1. Compared with the placebo and paroxetine, CHSGS will be as the same effective to the treatment for APE.
2. Compared with paroxetine alone, CHSGS and combination of drugs will be more effective due to its specific effects.
3. Investigate the influence of paroxetine or/and CHSGS capsule treatment on the brain activities of patients with APE compared with that of placebo treatment by fMRI and analyze the possible correlations between the changes of cerebral activity and the improvement of clinical variables in each group so as to explore how the paroxetine and CHSGS capsule manages depression by modulating brain function to treat APE.

## Methods

### Study design

**FIGURE 1:**
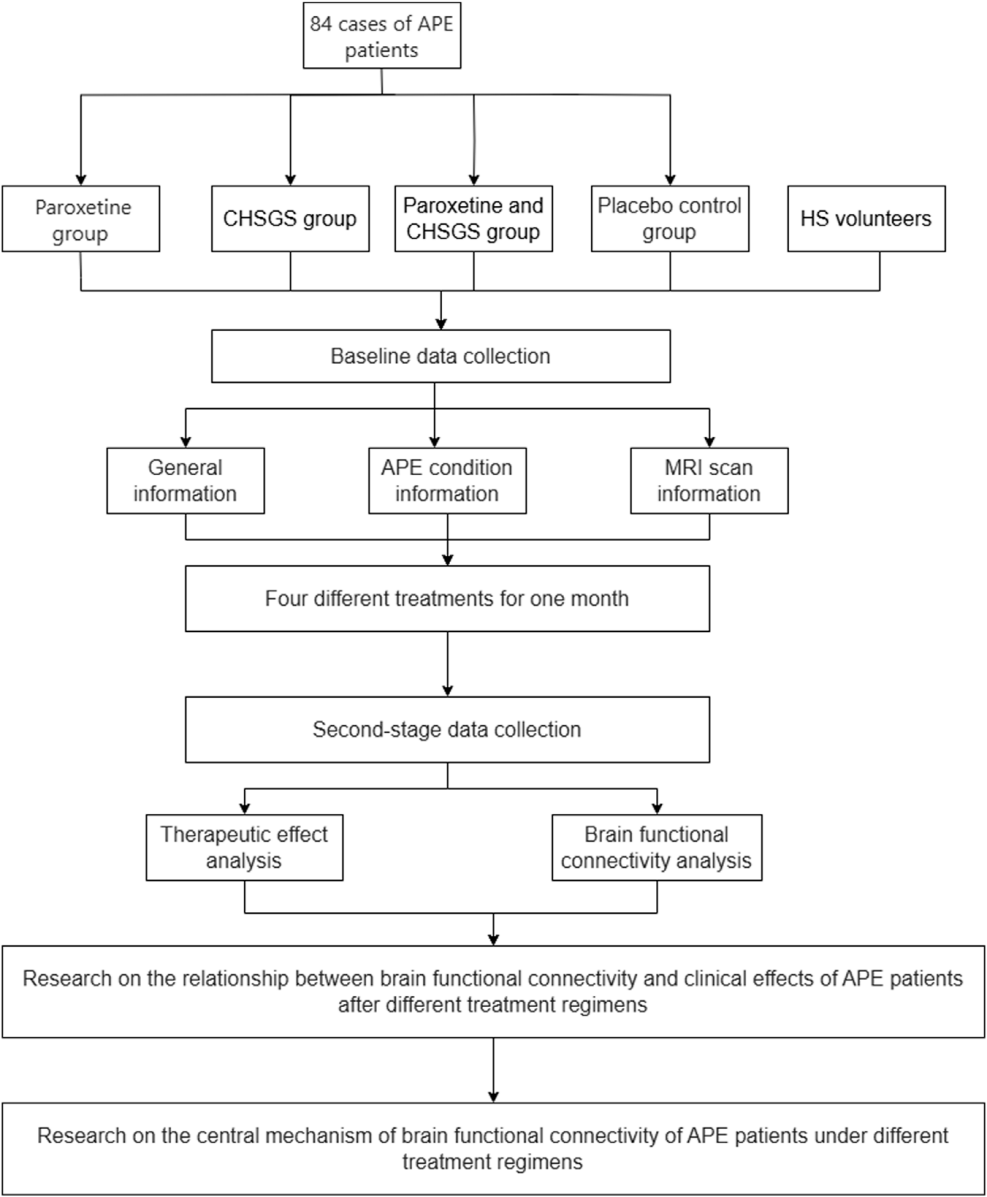
The flowchart of the study protocol.

This study will be a blinded interventional trial with four parallel groups and a natural control group. The study will be conducted in accordance with the Declaration of Helsinki and follows the Consolidated Standards of Reporting Trials statement for randomized trials, as well as the Standard Protocol Items: Recommendations for Interventional Trials (SPIRIT). The trial has been registered with the International Traditional Medicine Clinical Trial Registry (Registration No. ITMCTR2025000309) and Ethics Committee of Chongqing Hospital of Traditional Chinese Medicine (Registration No. 2023-ky-87), and will be proceeded in the Departments of Urology and Radiology at Chongqing Traditional Chinese Medicine Hospital in Chongqing, China. According to the minimum requirements for network analysis of functional neuroimaging studies, 15 subjects were needed in each group, and 21 subjects were included in each group to ensure the credibility of the study results, taking into account the unavailability of data due to patient dropout and head movement during scanning (Desmond and Glover, 2002). Through previous studies and in combination with the actual situation of the project (Rodríguez Del Águila and González-Ramírez, 2014), a total of 84 subjects were included in this study, and they will be equally divided into the following four groups: paroxetine group; CHSGS group; CHSGS + paroxetine group; placebo group. In addition, we will recruit 15 healthy volunteers as natural control group (Figure 1).

### Recruitment

Participants will be recruited from patients with APE who met the inclusion criteria in the urology clinic of Chongqing Traditional Chinese Medicine Hospital. Because it is not currently standardized, three senior physicians with a concurrent diagnosis of APE according to the ISSM definition of acquired premature ejaculation (a clinically significant and troubling reduction in ejaculatory latency time, usually about 3 min or less) are required for inclusion in this trial (Salonia et al., 2021). Eligible participants will sign the informed consent form before randomization.

### Participants

#### Inclusion criteria

1. Males whose age were between 18 and 70 years
2. Meet the minimum criteria for APE
3. PE short form (PEP) was compatible with acquired premature ejaculation
4. International Index of Erectile Function-5 (IIEF-5) is greater than 21
5. Sign the informed consent form.

#### Exclusion criteria

Although it meets the above diagnostic and inclusion criteria it belongs to:

1. Primary premature ejaculation patients
2. Merge with other serious systemic diseases or mental or neurological disorders
3. Patients without a fixed sexual partner or stable lifestyle
4. Spouses who have serious systemic illnesses and cannot cooperate.
5. Other conditions deemed unsuitable for participation by the researcher, which may lead to adverse consequences.

#### Removal criteria

1. Cases in which subjects were found after enrollment not to meet the inclusion criteria
2. Use of other medications related to the disease under study
3. Those who, after enrollment, were not treated in accordance with the provisions of this study
4. Cases in which there was no evaluable record of any post-treatment period.

#### Dropout criteria

1. The occurrence of serious adverse events, complications and special physiological changes, it is not suitable to continue to receive the trial
2. The trial process of self-withdrawal
3. Due to a variety of reasons caused by the end of the course of treatment, namely, withdrawal from the trial, lost to visit the case
4. Incomplete information, affecting the effectiveness and safety of the judgment.
The reason for removal should be recorded in all cases of exclusion or detachment. Any adverse reactions during treatment are included in the adverse reaction statistics.

### Randomized

Eligible participants will be randomly allocated to one of four groups in a 1:1:1:1 ratio: the paroxetine group, CHSGS group, CHSGS + paroxetine group, or placebo group. Randomization will be performed using SAS V.9.3 software, with sequences generated by an independent third party not involved in participant management, outcome assessment, or data analysis. Group assignments (denoted as A, B, C, or D) will be sealed in sequentially numbered, opaque envelopes prepared by a researcher unaffiliated with the trial. These envelopes will remain securely stored in numerical order until the study concludes to ensure allocation concealment. Access to the randomization codes will be restricted until final data analysis to maintain blinding integrity.

### Blinding

To ensure blinding in this study protocol, double - blind tests will be employed. Specifically, sham paroxetine and sham CHSGS capsules will be prepared. The Pharmacy Department of the Hospital of Chongqing Traditional Medicine Hospital will handle and label the medications in a special way. This is to guarantee that both the patients and the practitioners involved in the study remain completely unaware of the identity of the treatment being administered. Moreover, all practitioners are prohibited from communicating with the participants about the information related to this trial.

### Intervention

We prepared two colors of capsules, red capsules three times a day and yellow capsules once a day. Within the different groups, the contents of the different capsules were different.

### CHSGS group

Patients will be provided three-daily treatment with 5 g CHSGS added to the 5 red capsules and one-daily treatment with 5 g mixed starch powder added to the yellow capsule each time until 4 weeks.

The CHSGS preparation contained the gathering of the following components: 10 g Chaihu 12 g Shaoyao 12 g Zhiqiao 6 g Zhigancao 10 g Chenpi 10 g Chuanxiong and 15 g Xiangfu (Fan et al., 2023). They are made into a gel bags 100 pills per bottle made by the Pharmacy Department of Chongqing Traditional Chinese Medicine Hospital to meet the quality requirements of the Pharmacopoeia of the People’s Republic of China (2015 edition). The individual components of CHSGS, underwent decoction in boiling water for 30 min. After that, they were concentrated and then vacuum - dried to form a paste. Subsequently, these pastes were combined to create a new paste with a concentration of 8 g of crude extracts per gram. Next, starch powder was added to a container. The mixture was stirred thoroughly and then processed into capsules.

### Paroxetine group

Patients will be provided three-daily treatment with 5 g mixed starch powder added to the 5 red capsules and one-daily treatment with 30 mg paroxetine added to the yellow capsule each time until 4 weeks.

Dapoxetine and paroxetine are both 5-HT reuptake inhibitors for the treatment of premature ejaculation, however, dapoxetine is not recommended for daily administration, so that it is therefore difficult to observe its effects on the central nervous system. Also a trail showed that an on-demand dose of 30 mg dapoxetine is no more effective than the currently prescribed paroxetine treatment (Simsek et al., 2014). So we choose paroxetine as the positive control group as well as the treatment group. Smashed paroxetine will be mixed with starch powder and stir well into a container in order to be made into capsule.

### CHSGS + paroxetine group

Patients will be provided three-daily treatment with 5 g CHSGS added to the 5 red capsules and one-daily treatment with 30 mg paroxetine added to the yellow capsule each time until 4 weeks.

### Placebo group

Patients will be provided three-daily treatment with 5 g mixed starch powder added to the 5 red capsules and one-daily treatment with 5 g mixed starch powder added to the yellow capsule each time until 4 weeks.

It is well - recognized that placebos serve as the optimal comparator in clinical controlled trials. Consequently, we incorporated this placebo and standardized its characteristics. The placebo components were obtained from the Chongqing Traditional Chinese Medicine Hospital. The materials of placebo are starch and place the mixed starch powder into a container, add honey and stir well to make it into capsule. The above materials made shared the same character with the CHSGS capsule and paroxetine in terms of appearance, weight and taste.

### MRI scan

A GE3.0T magnetic resonance scanner (General Electric Company, America) at the Chongqing Traditional Chinese Medicine Hospital and a head orthogonal coil were used for 2 scans before and after treatment. A single excitation gradient echo planar imaging sequence was used for acquisition. The parameters were: TR/TE: 2000 m/30 m, FOV: 240 mm, matrix size: 64 × 64, flip angle: 90°, in-plane resolution: 3.75 mm × 3.75 mm, slice thickness: 5 mm thick with no gaps, and 32 sagittal slices.

### Outcome assessment

Independent assessors, who received standardized training prior to study involvement and remained blinded to group allocations throughout the trial, will evaluate all clinical outcomes. Comprehensive participant data - including both completers and those who discontinued intervention - will be systematically documented in electronic case report forms (CRF), ensuring complete tracking of all enrolled subjects.

### Primary outcome

Drawing on a range of clinical studies, the assessment will be carried out on all patients. Specifically, for patients with APE (Premature Ejaculation), the mean changes from the baseline (the day when the patient commences the protocol) to the end of the observation period (the day when the protocol concludes) will be evaluated in two aspects: the brain MRI and the intravaginal ejaculatory latency time (IELT) measured using a stopwatch, which has been extensively applied and is recommended as a primary outcome measure in clinical trials for APE.

### Secondary outcome

In terms of the assessment of symptom severity of APE for all the participants in this study, the secondary outcome will be based on the premature ejaculation profile (PEP) and Chinese Index of Premature Ejaculation (CIPE). In order to assess the patients’ TCM evidence and the degree of improvement, this trial also introduced the TCM syndrome score as a secondary outcome.

### Adverse events

During the trial, observers will properly estimate and record adverse events (AEs) associated with paroxetine treatment, including but not limited to vertigo, headache, and blurred vision. A specialized practitioner, who is not involved in clinical data analysis, will manage these AEs within 24 h. In the event that severe AEs occur, the principal researcher will make the final decision on whether to terminate the trial.

All data related to the participants during the study, as well as details of related and unexpected AEs (such as the time of occurrence, AE severity, and suspected causes), will be collected and documented in the Case Report Form (CRF). Depending on the severity of the AEs, the measures can range from providing symptomatic treatment to submitting the case to the Research Ethics Committee within 48 h.

### Quality control and data collection

Since any nonstandard or biased input of clinical data can significantly influence the bias of results, two practitioners, whose roles are to assess the treatment effect and data authenticity, will independently collect data using case report forms and original brain MRIs. The data thus gathered will be input into a dedicated computer. This entire process is designed to maximize the reliability and safety of all the data. To ensure the quality of the study, all practitioners involved must hold an official license and have at least 2 years of experience in protocol study and clinical practice.

### Data analysis

#### Clinical variables analysis

Two blinded evaluators will conduct the analysis of clinical data using SPSS 22.0 (SPSS Inc., Chicago, IL). Missing data analysis will be carried out in accordance with the intention - to - treat (ITT) principle, taking into account the baseline characteristics.

The clinical scores of the subjects will be examined through Pearson correlation analysis. All clinical data in this study will be presented in the following ways:

1. Continuous measurement data will be presented as the mean ± standard deviation (SD), along with the mean, SD, median, and interquartile range.
2. Categorical data will be presented as counts and percentages.

The following analysis tools will be employed: the Cochran - Mantel - Haenszel (CMH) test or nonparametric test, an independent samples t - test, and a two - sided test. Additionally, analysis of covariance, covariance analysis, generalized estimating equations, chi - square ( $x^{2}$ ) tests, and linear regression will be applied to all available and appropriate data. A P - value <0.05 will be considered statistically significant.

### MRI data analysis

Data preprocessing was carried out using the Statistical Parametric Mapping software package (Statistics Parameter Mapping 12.0, SPM12) (http://www.fil.ion.ucl.ac.uk/spm) (Tavares et al., 2020) and the Data Processing Assistant for Resting - State fMRI (DPARSF) based on the Matlab R2014b platform (Mathworks Inc., Sherborn, MASS). The preprocessing mainly included removing the first 10 time points, slice timing correction, head motion correction (Realign), spatial normalization, detrending, and noise filtering.

### Whole - brain functional activity regional homogeneity analysis

#### Regional homogeneity calculation

The Regional Homogeneity (ReHo) method was employed (Ji et al., 2020), assuming that there were similar changes in the same time series between the selected voxel and its adjacent voxels. In this study, the regional homogeneity of each voxel and its 26 adjacent voxels in the same time series was calculated point - by - point using the DPARSF software package. The Kendall’s Coefficient Concordance (KCC) was used to represent the ReHo value of the selected voxel and its neighboring voxels in the same time series, and a whole - brain ReHo map was generated for each subject (Zhu et al., 2020). Then, non - brain tissues were removed using a whole - brain template, and only the voxels within the template were included in the next step of analysis. To standardize the ReHo values, the ReHo value of each voxel was divided by the mean ReHo value of the whole brain. To ensure that the image data had the properties of a random Gaussian field to meet the statistical assumptions of SPM, spatial smoothing was performed after calculating the ReHo values. The data were smoothed using a Gaussian kernel function with a Full - Width at Half - Maximum (FWHM) of 6 mm to reduce spatial noise and minimize errors introduced during the spatial normalization process.

### Regional homogeneity analysis

SPM12 was used to conduct a regional homogeneity analysis of the resting - state imaging data. First, a paired t - test was used for the comparative analysis of the ReHo images of the APE subjects in each group before and after treatment. Meanwhile, to avoid noise data outside the brain tissue in the results, BrainMask_61 × 73 × 61. img was selected as the whole - brain mask file. The statistical threshold was set at P < 0.005, and the minimum activation cluster was required to be larger than 20 voxels. The results of the regional homogeneity changes in the brain regions before and after treatment were obtained respectively.

### Data monitoring and management

The Data Monitoring Committee will oversee safety and data monitoring and provide recommendations for trial design adjustments without any conflicts of interest. The Department of Urology at Chongqing Hospital of Traditional Chinese Medicine will ensure the quality of informed consent, recruit eligible participants, implement intervention measures, and manage data. Designated personnel will be responsible for collecting Case Report Forms (CRFs), as well as data transmission and analysis. Investigators and the principal investigator will be responsible for retaining all records, while the data center will store anonymized CRF data. Electronic data will be stored on password-protected computers, and all paper data will be kept in secure filing cabinets. Meanwhile, individual participant data can be accessed via the ResMan platform (Table 1).

**TABLE 1:** Standard protocol items.

Study Protocol Period							
	Enrolment	Distribution	Post-distribution(week)				
Time point			1	2	3	4	5
Enrolment:							
Eligibility screen	x						
Informed consent	x						
Demographics	x						
Allocation		x					
Intervetion:							
Paroxatine			x	x	x	x	
CHSGS			x	x	x	x	
Paroxatine+CHSGS			x	x	x	x	
Placebo			x	x	x	x	
Assessment:							
ZYZHPF	x					x	
PEP	x					x	
IELT	x					x	
CIPE	x					x	
MRI	x					x	
Adverse events			x	x	x	x	Call-back

## Discussion

In this protocol study, qualified participants are requested to adhere to their existing living habits and sexual behaviors. Particular attention should be paid to diet, exercise, and sleep quality. Simultaneously, they must complete relevant questionnaires during the entire observational period. To meet the requirements of the Ethical Code and minimize performance - induced biases, all other clinical procedures remain unchanged.

Based on current records and the results of recent randomized controlled trials, paroxetine has a lasting effect and is a favorable option for both patients and their partners in the treatment of APE (Zhang et al., 2019). Nevertheless, the central mechanism underlying its action remains unclear. Given the crucial role of the brain in APE, we have devised a randomized controlled MRI trial to uncover the central mechanism by which paroxetine treats APE (Gao et al., 2022). In this study, we will compare the alterations in brain function following the administration of three interventions: paroxetine, the CHSGS capsule, and combination therapy.

Premature ejaculation is a common male sexual dysfunction disorder, which is closely related to the central nervous system (Waldinger, 2006). In terms of neurotransmitters, an imbalance of serotonin, dopamine, and others can affect ejaculation. For example, abnormalities in serotonin - related receptors, an increase in glutamate concentration in the thalamus, and a decrease in GABA receptor expression are all associated with premature ejaculation (Andersson and Abdel-Hamid, 2011). There are many structural and functional abnormalities in brain regions such as the thalamus, frontal lobe, and somatosensory cortex. These include changes in the gray matter volume, local morphology, and functional connectivity of the thalamus, alterations in the thickness of the frontal lobe, gray matter volume and functional connectivity of specific regions, as well as abnormal related indicators in the somatosensory cortex. In addition, the thalamus - frontal lobe - somatosensory cortex circuit is also abnormal, such as a decrease in functional connectivity in some regions and a reduction in short functional connectivity density (Wu et al., 2024). These changes in the central nervous system collectively influence the occurrence and development of premature ejaculation (Chen et al., 2020). Unfortunately, however, much of the current research on premature ejaculation has focused on lifelong premature ejaculation, while relatively little research has been done on acquired premature ejaculation (Lu et al., 2020). Therefore, we hope to reveal the central nervous mechanism of acquired premature ejaculation and the intervention mechanism of Chinese medicine for acquired premature ejaculation (Xu et al., 2024).

In order to guarantee the reliability of the result of this study, we adopt the quality key point control as follows:

Subject Selection Criteria: For the purpose of baseline homogeneity, the patients in this study are restricted to those aged between 18 and 70 years old.

Sample Size Determination: To achieve stable statistical power, the sample size is carefully planned. In each group, 21 patients will be included for clinical evaluation, and 15 healthy volunteers will be included for the central mechanism study. The latter group will also undergo MRI scans.

MRI Scan Protocol: All MRI scans will be conducted in the afternoon, using the same scanner and operated by the same person. During the scanning process, all participants are required to remain relaxed, keep their eyes closed, and wear a birdcage head coil filled with sponge material. They must also stay still to minimize the impact of head movement. The scanning room is maintained with noise levels below 150 dB, a temperature range of 18 °C–22 °C, and a humidity level higher than 60%.

In conclusion, this study is conducted in the purpose of supporting the concept of brain MRI alteration in APE with paroxetine or CHSGS capsule. We expect that the results of this trial can provide both an evidence-based treatment option for patients suffering from APE, an enhanced level of evidence on which central mechanism research and to provide an innovation of the clinical treatment in APE.
